# Elastic and Ultradeformable Liposomes for Transdermal Delivery of Active Pharmaceutical Ingredients (APIs)

**DOI:** 10.3390/ijms22189743

**Published:** 2021-09-09

**Authors:** Eliana B. Souto, Ana S. Macedo, João Dias-Ferreira, Amanda Cano, Aleksandra Zielińska, Carla M. Matos

**Affiliations:** 1 CEB—Centre of Biological Engineering, University of Minho, Campus de Gualtar, 4710-057 Braga, Portugal; 2 Department of Pharmaceutical Technology, Faculty of Pharmacy, University of Coimbra, Pólo das Ciências da Saúde, Azinhaga de Santa Comba, 3000-548 Coimbra, Portugal; j.dias.ferreira@outlook.pt (J.D.-F.); aleksandra.zielinska@igcz.poznan.pl (A.Z.); 3 Faculty of Health Sciences, Universidade Fernando Pessoa, Praça 9 de Abril, 349, 4249-004 Porto, Portugal; anatmacedo@hotmail.com; 4 LAQV, REQUIMTE, Department of Chemical Sciences—Applied Chemistry Lab, Faculty of Pharmacy, University of Porto, Rua Jorge de Viterbo Ferreira 228, 4050-313 Porto, Portugal; 5 Department of Pharmacy, Pharmaceutical Technology and Physical Chemistry, Faculty of Pharmacy and Food Sciences, University of Barcelona, 08028 Barcelona, Spain; acanofernandez@ub.edu; 6 Institute of Nanoscience and Nanotechnology (IN2UB), University of Barcelona, 08028 Barcelona, Spain; 7 Institute of Human Genetics, Polish Academy of Sciences, Strzeszyńska 32, 60-479 Poznań, Poland

**Keywords:** skin inflammatory diseases, psoriasis, psoriasis vs. atopic dermatitis, biodegradable nanoparticles, microneedles, clinical trials

## Abstract

Administration of active pharmaceutical ingredients (APIs) through the skin, by means of topical drug delivery systems, is an advanced therapeutic approach. As the skin is the largest organ of the human body, primarily acting as a natural protective barrier against permeation of xenobiotics, specific strategies to overcome this barrier are needed. Liposomes are nanometric-sized delivery systems composed of phospholipids, which are key components of cell membranes, making liposomes well tolerated and devoid of toxicity. As their lipid compositions are similar to those of the skin, liposomes are used as topical, dermal, and transdermal delivery systems. However, permeation of the first generation of liposomes through the skin posed some limitations; thus, a second generation of liposomes has emerged, overcoming permeability problems. Various mechanisms of permeation/penetration of elastic/ultra-deformable liposomes into the skin have been proposed; however, debate continues on their extent/mechanisms of permeation/penetration. In vivo bioavailability of an API administered in the form of ultra-deformable liposomes is similar to the bioavailability achieved when the same API is administered in the form of a solution by subcutaneous or epi-cutaneous injection, which demonstrates their applicability in transdermal drug delivery.

## 1. Introduction

The skin is the largest organ of the human body, weighing up to 16% of total body weight, and consisting of a complex structure of several strata that form a barrier between the body and the external environment [1,2]. This barrier is not completely waterproof and allows gas, heat, and fluid exchanges with the outside. Its main function is the protection of the body against microbial, chemical, mechanical, thermal, and radiation injuries [3,4].

The skin is split into three layers—the epidermis, the dermis, and the subcutaneous layer [3,4,5]. From the inside to the outside, the first layer is the subcutaneous layer that comprises subcutaneous fat (hypodermis), followed by the dermis, which contains the connective tissue and, finally, by a cell layer without vascularization, called the epidermis [4,5].

The dermis is about 3–5 mm thick and is composed of fibrous proteins, mostly of collagen and elastin, and an interfibrillar gel of glycosaminoglycans, salts, and water. It is in the dermis that structures, such as lymphatic and blood vessels, nerve endings, hair follicles, sebaceous glands, and sweat glands are found. It also has hair follicles and sweat glands that are appendages that extend to the surface of the skin, which makes them a possible route of entry for active pharmaceutical ingredients (APIs) [6].

The epidermis is not vascularized; thus, the nutrients and metabolic products are diffused through the dermoepidermal junction so that the dermis maintains its vitality. The epidermis is further subdivided into five strata—the germinative stratum or basal stratum, the spinous stratum, the granular stratum, the lucid stratum, and the stratum corneum, from the inside to the outside. Keratinocytes are the fundamental cells of the skin that undergo proliferation, differentiation, and keratinization, from the inside to the surface of the skin, where they become the corneocytes of the stratum corneum. The epidermis is also made up of other cells, such as melanocytes involved in melanin synthesis, Merkel cells responsible for sensory functions, and Langerhans cells–dendritic cells with immune function. The stratum corneum is composed of 15–20 layers of corneocytes that reach a thickness of 10 to 15 µm that can reach up to 40 µm in thickness when the skin is hydrated. The main constituents of the stratum corneum are non-viable, flattened cells, filled with fibrous proteins and keratin, produced by the epidermis [3,6,7].

**Figure 1:**
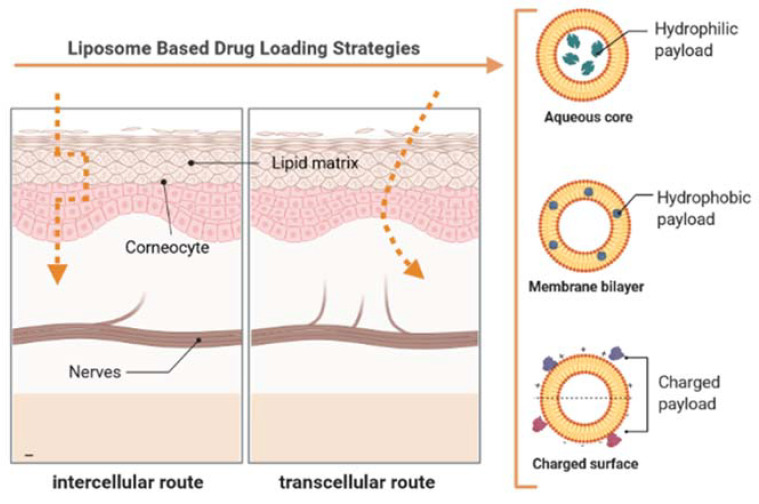
Schematic representation of the compound permeation pathways on the skin (on the left). Permeation occurs mainly through the intercellular and intracellular pathways. The elastic and ultra-deformable liposomes penetrate the skin essentially through the intercellular route since the phospholipid bilayer is flexible and allows its deformation to cross the intercellular spaces. Schematic representation of an ultra-deformable liposomal system to administer APIs (on the right).

The stratum corneum is continually renewed and is the main barrier that prevents dehydration of the inner strata and at the same time limits the access of water and compounds from the outside. Thus, the absorption of APIs is also limited. Even so, it is thought that the main permeation pathways are through the appendages of the skin (adnexal route) and via the transepidermal route [3,6]. When APIs penetrate through the adnexal route, the permeation is made by the sweat glands, sebaceous glands, and hair follicles. The transepidermal pathway is a pathway through which the compounds pass through the skin through the intact stratum corneum. This route is further subdivided into two other routes. The first, the intercellular pathway, consists of a continuous permeation of the compounds between cells through the intercellular lipids, and in the second, the intracellular pathway, there is a permeation of the compounds in the keratinocytes, and later, diffusion to the intercellular lipids (Figure 1). In general, the compounds penetrate the skin through the three pathways according to their physical–chemical characteristics; however, the intercellular pathway is the main pathway of penetration [5,6].

As the stratum corneum is the main barrier for transdermal mass transport through the skin, the therapeutic plasma levels of APIs are compromised; thus, distinct approaches have been developed to improve the transdermal absorption of APIs, which include vesicles, such as liposomes [8], niosomes [9], and ethosomes [10], which can be combined with invasive techniques e.g., microneedles [11,12], iontophoresis [13], electroporation [14], and ultrasounds [15]. The application of the latter onto the skin is, however, recommended as a fast and reversible process, so that no toxic effects on epidermal and dermal cells are seen [5].

The skin has its advantages for the administration of APIs since it allows non-invasive delivery of therapeutic agents, minimizing pain and the risk of infection; therefore, resulting in the patient having higher compliance to the treatment, allowing both systemic and local treatments. The drug delivery through the skin avoids the first-pass metabolism (described for the oral route), theoretically leading to a higher bioavailability of the therapeutic compounds [16,17]. The potential of liposomes to modify the release profile of loaded APIs has also been pointed out as an advantage [18].

The transdermal administration of APIs has relevant advantages over other routes that include: (i) the possibility of local action by APIs, reducing their side effects in general; (ii) systemic administration of APIs that overcomes the effect of the first hepatic passage, avoidance of gastrointestinal degradation of APIs, and pain from intravenous or subcutaneous injections. In this work, we discuss the use of liposomes, their classification, composition, advantages, and limitations in delivery APIs through the skin.

## 2. Liposomes for Transdermal Administration of APIs

### 2.1. Classification of Liposomes

Liposomes are spherical vesicles, consisting of one or more concentric bilayers of phospholipids, surrounding an aqueous core. Since their discovery, in the 1960s, they have been studied as APIs transport systems, with several pharmaceutical formulations based on these systems already reaching the market [19,20]. Due to their biphasic structure, liposomes are capable of encapsulating both hydrophilic APIs (dissolved in the aqueous core) and lipophilic (distributed in the lipid membrane). Amphiphilic compounds can interpenetrate the membrane, while charged compounds can be associated with the surface of liposomes [21]. Liposomes are highly versatile and changeable vehicles and can be used in modified-release formulations, for targeting specific organs, or as protectors of molecules that are sensitive to light, pH, or to aqueous medium. They are biocompatible, biodegradable, have reduced toxicity, and have the ability to change the pharmacokinetic profile of loaded APIs [22]. Classic liposomes are made up of lipids, usually phospholipids, of natural or synthetic origin, such as phosphatidylcholine, phosphatidylethanolamine, phosphatidylserine, phosphatidylinositol, and may include other lipids, such as cholesterol, cardiolipin, or sphingomyelin, depending on the desired characteristics, as membrane fluidity, phase transition temperature, membrane potential, or degree of hydration. Table 1 depicts the classification of the liposomes concerning the physical structure [23]. There are also other names sometimes found in the literature that refers to preparing the vesicles: reverse phase evaporation vesicles (RPEV), reconstituted dry vesicles (RDV), and multilamellar vesicles (MV).

### 2.2. Classic Liposomes vs. Elastic/Ultra-Deformable Liposomes

The first liposome-containing preparation for transdermal administration of APIs was launched in the early 1990s, composed of econazole for antimycotic treatments [24]. Previous studies on liposomes for topical application of APIs described that the penetration of APIs reached the vascular dermis, but that the liposomes, due to their large size, would not be able to reach the blood circulation; thus, functioning as a reservoir for APIs. The liposomes used were mostly multilamellar vesicles (MLs); however, the liposomes found in the dermis were unilamellar, which was an indication that the MVs would lose their outermost layers during skin penetration [25].

Other attempts to use this vehicle in transcutaneous preparations followed, but in general, with little success, since conventional liposomes do not seem to penetrate to the deepest layers of the skin, remaining confined to the most superficial space of the epidermis [26].

Some physical–chemical characteristics seem to facilitate the penetration of conventional liposomes, such as application in the gel phase, use of cutaneous lipids for the preparation of vesicles, increased membrane fluidity by decreasing the amount of cholesterol, or changing properties, such as size, charge, and lamellarity [27].

However, these liposomes have several limitations, such as (i) they are not suitable for transdermal administration, since they cannot reach the deeper layers of the skin, being accumulated on the surface of the stratum corneum; and (ii) when transdermal penetration occurred, plasma levels reached fall well below the concentration needed to achieve therapeutic effect [28].

Over the past 15 years, intensive research to overcome the limitations of transdermal administration of molecules resulted in the introduction and development of a new class of extremely elastic and ultra-deformable liposomes.

In addition to their structure or method of preparation, the nomenclature of liposomes can indicate their composition or special characteristics. Taking into account these aspects, they can be differentiated in (i) transfersomes (Transfersomes^®^), the term ’transfersome‘ was registered by the German company IDEA AG (Munich, Germany), and means ’transporting body‘: it derives from the Latin word ’transferre‘ (transport) and the term Greek ’soma‘ (body) [29]. They are highly deformable liposomes, used to increase the bioavailability of APIs through the skin. (ii) Niosomes, which are vesicles formed by nonionic amphiphilic agents, such as Span or Tween, in replacement of phospholipids, as they have greater chemical stability. Niosomes have been used for topical delivery of APIs; (iii) ethosomes, which are vesicles formed by phospholipids, ethanol, and water; the interdigitation of alcohol molecules in the phospholipid best rate makes lipids less densely packed and more malleable, maintaining stability. In this way, they are more effective in transporting APIs through the skin; (iv) cerasomes, colloidal particles made up of the vesicle with the bi-stratus coated with a surface with polyorganosiloxane, which gives it greater stability than conventional liposomes; and finally (v) polymersomes, which are vesicles formed by self-aggregation of high molecular weight copolymers [30]. Since polymersomes are formed by synthetic macromolecules, they are more stable and allow greater control of chemical and structural properties.

Den Bergh et al. developed elastic vesicles, which, instead of being formed by phospholipid bilayers, were formed by bilayers of surfactants, such as sucrose laurate ester and octoxyethylene laurate ester [31,32].

The concept of ultra-deformable liposomes was first introduced in 1992 by Cevc and collaborators [33]. Since then, many researchers have developed ultra-deformable liposomes to administer APIs transdermally [16,34]. Ultra-deformable liposomes are biocompatible and biodegradable since they are formed from natural phospholipids, similar to conventional liposomes. They generally achieve high encapsulation efficiencies that can reach 90% in the case of lipophilic APIs, but also allow the encapsulation of hydrophilic APIs and molecules of low and high molecular weight. In this way, they contribute to an increase in the APIs half-life and allow their slow release over time. Their elastic nature allows these liposomes to be able to penetrate more deeply into the skin when compared to conventional non-elastic liposomes [28,35].

### 2.3. Composition of Ultra-Deformable Liposomes

Ultra-deformable liposomes are formed by phospholipids and a surface activator that has a single chain with a high radius of curvature and, for this reason, destabilizes phospholipids, forcing a redistribution of amphiphilic molecules, causing an increase in the flexibility of the lipid bilayer [16,28,36,37,38]. Phosphatidylcholine, which is the most abundant lipid of the cell membrane, is the most commonly used in the production of transfersomes, thereby contributing for their high skin tolerance and for the decrease of side effects, irritation, and/or hypersensitivity [39]. These compounds are generally surfactants, such as sodium cholate, sodium deoxycholate, polysorbates. The presence of surfactants in the liposomes allows deformation of the liposomes without breaking them, making the phospholipid bilayer more fluid and flexible, thus allowing the liposomes to cross the intercellular spaces of the stratum corneum that have one-tenth the diameter of the liposomes without significant losses of APIs [6,16,28,34,40,41,42]. The addition of agents that are toxic and biodegradable do not cause adverse reactions, such as skin irritation or toxicity, which leads to the high topical tolerance of these formulations.

El Zafaarany et al. tested the influence of different surfactants on shape, size, encapsulation efficacy, strain rate, and in vitro release of sodium diclofenac encapsulated in liposomes [43]. The results showed that the liposome formulation significantly increased the amount of drug that was placed on the surface of the stratum corneum and that permeated the skin, compared to the marketed product. The study also proved that the transepidermal flow and the prolonged release of sodium diclofenac were dependent on the type of surfactant and the method of preparation, making it possible to optimize ultra-deformable liposome formulations.

Like conventional liposomes, elastic/ultra-deformable liposomes are nanometric vesicles that are sensitive to mechanical stress, quickly changing their shape to penetrate more deeply into the skin. A new approach to increase skin permeation of these liposomes includes the use of peptides (such as Tat, YARA, WLR, and R9) that interact with the stratum corneum lipids, causing the internalization of the liposomal system via macropinocytosis [44]. Melatonin has been proposed to increase skin permeation of liposomes [45].

## 3. Preparation and Analysis of Ultra-Deformable Liposomes

The methods for the preparation and characterization of ultra-deformable liposomes do not differ greatly from those commonly used for conventional liposomes.

The classic process of preparing liposomes, first described by Bangham et al. is the method of hydrating the lipid film [46]. In this method, a lipid solution prepared with an organic solvent (usually chloroform and/or methanol) is evaporated to form a thin, homogeneous film on the walls of a glass flask. The film is then hydrated with an aqueous solution, with spontaneous formation of vesicles.

By mechanical treatment, the formed MVs can be transformed into other structures, such as SUVs and LUVs, with the most widespread processing methods being the use of ultrasound and extrusion. The use of ultrasound, which was the first method used to homogenize dispersions of MV, can be carried out in a water bath or with a titanium tip, which is used when greater energy is required [47]. The sonication process can be harmful to many compounds, including lipids. The use of the water bath has the advantage of not being so aggressive, but it requires a longer treatment time.

The extrusion is based on the forced passage of the dispersions of MVs through filters of well-defined pore diameter under high pressure (approximately 100 atm) carried out with an inert gas. The lipids must be extruded at a temperature above the phase transition temperature, and several passes through the filter are necessary to homogenize the size of the liposomes. Repeated extrusions decrease the number of lamellae and after approximately 10 passes through the filter, the liposomes are predominantly unilamellar. Its final diameter will depend on the pore size used and the characteristics of the lipid itself, but in general, diameters below 50–60 nm cannot be achieved [48].

Other methods widely used for the preparation of vesicles are injection methods, mainly the injection of ethanol, and the injection of ether. Briefly, a solution of lipid in the organic solvent is quickly injected into an excess of aqueous solution through a fine needle [49]. This procedure results in a very high proportion of SUVs in diameter of about 25–30 nm. The advantages of the method are simplicity and the absence of potentially harmful physical processing. The biggest disadvantage is the remaining solvent in the aqueous phase, which will have to be removed by dialysis or gel filtration.

Other methods, such as reverse phase evaporation and dehydration/rehydration, are less widespread. In reverse phase evaporation, the aqueous phase is emulsified in the presence of phospholipids dissolved in ethyl ether or in any other volatile and water-immiscible solvent. The evaporation of the solvent, under moderate vacuum, interrupted by strong agitations to break the gelled structures, produces the liposomes, fundamentally of the LUVs type.

The dehydration/rehydration technique involves the lyophilization of multilamellar or unilamellar liposomes, dispersed in an aqueous medium containing the APIs to be incorporated, followed by controlled rehydration, obtaining MVs with high incorporation efficacy. We can also consider the use of freeze/thaw cycles, a technique that allows to increase the efficiency of encapsulation of APIs and to improve the physical characteristics of the lipid membrane [50].

After preparation, ultra-deformable liposomes are characterized in terms of their physical and chemical properties and the effectiveness of APIs encapsulation, in a very similar way to what happens with conventional liposomes. Mostly used for topical administration, their evaluation in pharmacokinetic terms generally includes studies of permeation through the skin.

Thus, the physical–chemical characterization of transfersomes comprises (i) the size and zeta potential: parameters that can be obtained by photonic correlation spectroscopy or light scattering techniques, which also allow obtaining the degree of dispersion, a measure of the heterogeneity of vesicles; (ii) shape and lamellarity: can be obtained by transmission or scanning electron microscopy; (iii) the lipid composition: determined by different analytical techniques, such as high-resolution chromatography or spectrophotometry; and (iv) the phase transition temperature, assessed by differential scanning calorimetry, which also allows the determination of the enthalpy of the phase transition, representative of the degree of connectivity between phospholipids [51,52].

The encapsulation efficiency and the APIs release rate are fundamental parameters that must be considered in the development of these formulations. Techniques, such as ultracentrifugation, high-performance liquid chromatography (HPLC), or spectrophotometry after separation of the non-encapsulated compound and rupture of the vesicles with an appropriate solvent, can be used [52]. The release of APIs can be assessed using techniques, such as dialysis, evaluating the amount of APIs released by different analytical techniques [53].

Skin permeation studies can be performed using confocal laser microscopy. In this technique, transfersomes are labeled with a fluorescent molecule, such as a lipid derivative of rhodamine or fluorescein. This technique allows analyzing the histological organization of the skin and the penetration of the liposomes through the different strata. Incubation of the liposomes with the skin, human, or animal, or with an appropriate membrane can be achieved using Franz cells [27].

## 4. Permeation Mechanisms of Conventional vs. Ultra-Deformable Liposomes

Liposomes can perform several functions upon topical application. They allow increasing the amount of drug that is placed on the skin to reach the site of action while the systemic effects are reduced, thereby exhibiting a local effect A systemic effect can also be achieved in case of using, for example, the adnexal or intercellular route as a gateway for APIs [6]. The pathways by which liposomes permeate the skin are quite different. According to the permeation mechanism of free APIs, APIs can permeate the skin regardless whether they are loaded inside the vesicles or attached to the surface. The increased APIs penetration results from the vesicles dissolution in the skin and join the stratum corneum, forming structures with intercellular lipid lamellae, thereby promoting APIs’ penetration. In the mechanism of adsorption or fusion with the stratum corneum, the liposomes adsorb to its surface with the release of APIs directly on the skin. Otherwise, the vesicles fuse with the lipid matrix of the stratum corneum, increasing the diffusion of APIs within the skin. Phospholipids of liposomes lipid bilayer also act as penetration enhancers thereby promoting the partition of several molecules in the stratum corneum [6,41]. Although conventional liposomes can interact with the skin and increase the permeation of APIs, they cannot cross the healthy stratum corneum intact [41]. Ultra-deformable liposomes favor the penetration of APIs through the stratum corneum by two mechanisms. In the first, the liposomes act as vehicles for APIs, and pass through the stratum corneum in its intact form. Additionally, the vesicles also act as absorption enhancers, disturbing the lipid layer at the surface of the skin, crossing the stratum corneum, and modifying the structure of the intercellular lipid lamellae, promoting the penetration of the APIs through the stratum corneum [28,41].

In the first mechanism, liposomes penetrate the skin due to xerophobia (tendency to move away from dry places). Because of their elasticity, ultra-deformable liposomes are resistant to mechanical stress, being able to deform and cross the channels between the cells of the stratum corneum, crossing the epidermis, reaching the dermis and eventually, systemic circulation. Studies suggest that the two mechanisms can occur simultaneously, but in general, one predominates over the other, depending on the physical–chemical characteristics of the APIs (hydrophilic vs. lipophilic) and the composition of the liposomes [28,41]. Once applied to the skin, liposomes structurally modify the stratum corneum and it was possible to identify intact vesicles in the intercellular lipid regions. However, intact vesicles have not been identified in the deeper tissues of the skin. There is still a lively debate in the scientific community about the behavior of ultra-deformable liposomes and the question remains whether they can actually get through the skin without suffering degradation, behaving like true API transporters or if they only act as promoters of API permeation [38].

The transepidermal gradient of hydration is caused by the difference in water content between the surface of the skin (about 20%) and the deeper layers (about 100% in the epidermis), which prevents dehydration of the skin. All polar lipids attract water, resulting from an energetically favorable interaction between hydrophilic lipid residues and water. Consequently, when placed onto the surface of the skin, they tend to move from the application area, poor in water content due to dehydration, to the deeper strata that are rich in water [6,28]. This gradient is, then, the driving force for the transfer of the transfersomes along the strata of the skin. Transfersomes make their way between cells, passing the stratum corneum in places that offer less resistance to their passage [54].

The great capacity of transfersomes to deform is achieved at the expense of a feedback mechanism, which allows these vesicles to adapt their shape to the conditions of the surrounding environment, in an easy, fast, and reversible fashion [39]. This ability is related to the composition of these vesicles that are formed by mixtures of less soluble amphiphilic agents, which can form spheroidal or discoid aggregates, and more soluble, which can adopt a quasi-planar conformation. Therefore, when the vesicle is exposed to non-uniform local stress, this causes its elongation and the individual components begin to separate into different regions of the membrane, accumulating the most soluble in the places of greatest deformation [54].

## 5. Influence of Physicochemical Characteristics on Transdermal Administration

The release of APIs and the topical application of cosmetic ingredients is influenced by many factors. In general, these factors include the size of the molecules, the hydrophilic or lipophilic nature of the compounds, the type of formulation, the presence of absorption promoters, and the state of the stratum corneum. The success of liposomes for topical application is influenced by their lipid composition, size, elasticity, the load on the surface of the liposomes, the method of application, and the total lipid concentration [3,55]. Some authors claim that the permeation of liposomes can be improved when the lipid composition of the bilayer is heterogeneous, i.e., when several lipid domains with different flow characteristics coexist and modify the permeation properties of several loaded APIs [3]. The permeation of liposomes to the deepest layers of the skin under occlusive and non-occlusive conditions is also matter of concern. Cevc et al. concluded that ultra-deformable liposomes are more effective when applied under non-occlusive conditions [56]. Non-occlusive application is essential to create the transepidermal osmotic gradient and, consequently, penetration into the skin [3].

## 6. Applications of Ultra-Deformable Liposomes

Ultra-deformable liposomes have been widely used in recent years in various aspects of medicine. Their most common application has been as a transport system for molecules for cutaneous administration, such as proteins, peptides, genes, hormones, antihypertensive agents, anti-inflammatory drugs, corticosteroids, among others (Table 2).

The loading of APIs in liposomes was reported to be instrumental for several purposes, i.e., only allows modulating the pharmacokinetic profile of APIs but also protects APIs from enzymatic action and degradation at different pH values. The vast majority of peptides and proteins cannot be administered orally in conventional pharmaceutical forms, since they are large molecules and thus, hardly transported by the body and, when administered orally, suffer degradation in the gastrointestinal tract; therefore, their administration has always been injectable. These compounds were then encapsulated in ultra-deformable liposomes since their bioavailability in the skin is similar to that obtained by subcutaneous injection. The skin is a more appealing route of administration, as it is non-invasive and has Langerhans cells involved in the immune response [29]. Gupta et al. demonstrated that it is possible to obtain immunization against the toxoid tetanus antigen through the topical administration of liposomes [70]. The effectiveness of the systems was assessed by the collection of IgG antibodies in the serum. The primary and secondary immune responses were found to be synergistic when administering the commercial formulation containing the same dose of antigen by intramuscular route.

Montanari et al. obtained promising results when using ultradeformable liposomes activated by sunlight [71]. These photodynamic liposomes were developed to encapsulate zinc phthalocyanine for the treatment of *Leishmania braziliensis* infections. Zinc phthalocyanine has 20% anti-promastigote and anti-amastigote activities; however, when encapsulated in ultra-deformable liposomes, the percentages of inhibition were 100% and 80%, respectively. The authors also concluded that the transcutaneous penetration of zinc phthalocyanine was about 10 times higher when encapsulated in ultra-deformable liposomes compared to conventional liposomes, having a homogeneous distribution throughout the stratum corneum. Thus, the transepidermal route is a promising route for the treatment of *L. braziliensis*.

Maestrelli et al. addressed the complexing of anesthetics with their subsequent encapsulation in liposomes [72]. Benzocaine and butadiene were complexed with cyclodextrins and later encapsulated in ultra-deformable liposomes. Liposomes have been characterized in terms of their size, charge, shape, and encapsulation efficiency. The obtained results demonstrated not only an increase in the skin permeation of the APIs, but also an increase in the intensity and duration of the anesthetic in vivo effect.

Ahad et al. developed a liposomal system to encapsulate valsartan, an APIs used in the treatment of arterial hypertension [73]. The results obtained demonstrated a superior therapeutic effect carried out by ultradeformable liposomes when compared to conventional liposomes, due to an increase in the drug transepidermal flow, these liposomes being a suitable vehicle for the topical administration of antihypertensives.

Mishra et al. developed elastic liposomes for the transcutaneous immunization of Hepatitis B antigen [74]. Liposomes were characterized in terms of their size, encapsulation efficacy, elasticity, turbidity, stability, and in vitro release. Ex vivo and in vivo studies were also conducted. In vivo studies revealed that the transcutaneous immunization carried out by elastic liposomes induced exacerbated systemic and topical responses against the hepatitis antigen, having proved the potential of these systems as non-invasive vaccines [74]. Dubey et al. on the other hand, chose melatonin, since it has a short half-life, low molecular weight, and variable oral absorption, to develop a new form of transdermal application [45]. The loading of melatonin in elastic liposomes increased its cutaneous permeation compared to conventional liposomes and the free melatonin solution, approximately 5 and 12.3 times, respectively. These studies denote the versatility of liposomal systems in the encapsulation of different molecules with promising results about transepidermal administration. The analgesic ketorolac tromethamine was loaded in ultradeformable liposomes using Tween 80 as an edge activator, and tested ex vivo and in vivo [75]. The ex vivo permeation studies of the analgesic across pig ear skin by Franz diffusion cells resulted in a flux of 0.278 μg/cm^2^·h and a lag time of about 10 h anticipating the production of a drug depot in the skin to show a local effect. The in vivo study conducted on healthy human volunteers using a tape-stripping technique showed correlation between the total amount permeated, the penetration distance, and transepidermal water loss.

Bashyal et al. produced sodium cholate-incorporated elastic liposomes and sodium glycodeoxycholate-incorporated elastic liposomes, which were tested across porcine buccal tissues using confocal laser scanning microscopy [76]. The authors showed significantly enhanced the permeation of insulin with a 4.33-fold increase in the permeability coefficient compared to insulin solution.

Ex vivo transdermal permeation of diclofenac from a 1% liposomal gel formulation in excised human skin was compared to two emulsion gel formulations, 1.16% and 2.32% diclofenac diethylamine (equivalent to 1% and 2% diclofenac sodium) by Sacha et al. [77], highlighting a clinical advantage of the liposomal gel attributed to a higher transdermal permeability coefficient.

Despite the attention that lipid vesicles have attracted in recent years, it is generally accepted within the scientific community that classic liposomes have few advantages for the targeting of APIs through the skin, since they do not penetrate the deeper layers of the skin, becoming confined to the stratum corneum [28,34,37,38].

## 7. Conclusions

The vast majority of topical and transdermal formulations are composed of lipophilic and low molecular weight APIs, which cross the skin by passive diffusion. However, the permeation of hydrophilic APIs with a molecular size greater than 500 Da is very modest. Thus, there is a need to develop strategies aimed at the topical and transdermal administration of these molecules. Liposomes are nanosized lipid vehicles formed by phospholipid bilayers. These liposomes are not very stable and are not suitable for cutaneous administration since after topical application they accumulate on the skin surface, due to the rigidity of the lipid layers and undergo dehydration, culminating in their fragmentation. Then came ultra-deformable liposomes, patented under the name Transfersomes^®^, to overcome the limitations of conventional liposomes. The ultra-deformable liposomes are more flexible due to the introduction of surfactants in the phospholipid membranes, which destabilize the amphiphilic molecules, causing an increase in the fluidity of the membrane. Subsequently, elastic liposomes emerged, which, like ultra-deformable liposomes, are also more flexible than conventional liposomes, however, consisting of bilayers of surfactants. These two liposomal systems are susceptible to mechanical stress and change in their shape. These changes in shape allow them to permeate biological structures, such as the skin. The in vivo biodistribution of the ultra-deformable liposomes applied topically is similar to the biodistribution of the application by the epi-cutaneous route or subcutaneous injection of the same formulations. The conditions regarding the application of the formulations influence the penetration of these systems, which can thus be controlled to exert a strictly local effect.

## Figures and Tables

**Table 1:** Classification of the different types of liposomes based on their physical structure.

Type	Abbreviation	Size	Other Characteristics
Multilamellar Vesicles	MV	Diameter between 0.4 and 3.5 µm in diameter, with the average size being about 1 µm	Obtained by simply dispersing the phospholipids in the aqueous medium, and not subject to further processing. Each vesicle consists of several lipid lamellae (around five or more) arranged concentrically, including a fraction of the internal aqueous medium.
Large Unilamellar Vesicles	LUV	Diameter greater than 100 nm	Composed by a single layer.
Small Unilamellar Vesicles	SUV	Diameter between 25 and 50 nm	
Unilamellar vesicles of intermedian size	UVIS	Diameter between 50 and 100 nm	
Giant unilamellar vesicles	GiUV	Radius greater than 10 µm	
Oligovesicular vesicles	OVV	-	Structures formed by small vesicles incorporated in a larger one.

**Table 2:** Example of several APIs loaded in elastic/ultra-deformable liposomes for transdermal applications.

Drug	Therapeutic Indication	Observations	Reference
Bleomycin	Anti-tumoral	Increased dermal and epidermal permeation	[57]
Carvedilol	Skin carcinogenesis	EGF-induced neoplastic transformation of mouse epidermal JB6 P+ cells at non-toxic concentrations	[58]
Clotrimazole	Fungal infections	Increased skin permeation and inhibition of fungal growth	[35]
Corticosteroids	Anti-inflammatory	Decrease in the dose of corticosteroids needed to suppress edema	[59]
Cyclosporine A	Immunosuppression	Increased transepidermal flow	[60]
DNA	Gene therapy	Increased cell internalization and gene expression	[61]
Diclofenac	Joint anti-inflammatory	Increased skin permeation	[37]
Docetaxel	Anti-tumoral	Increased transdermal flow	[62]
Estradiol	Hormonal therapy	Increased transepidermal flow	[63]
Fluorouracil	Non-melanoma skin cancer	Co-loaded with resveratrol arrested cell proliferation in G1/S, modifying the action of 5-fluorouracil and increasing the activity of resveratrol	[64]
Heparin	Antiplatelet agent	Skin permeation	[65]
Hepatitis B antigen (anti-HBsAg)	Hepatitis B virus	Increased immune response	[66]
Insulin	Diabetes mellitus type 1	Increased transepidermal flow	[67]
Ketoprofen	Anti-inflammatory	Increased drug concentration in muscles	[68]
Methotrexate	Psoriasis	Increased permeation and accumulation in the dermis and epidermis	[42]
Resveratrol	Antioxidant	Resveratrol-loaded liposomes were formulated in a topical cream retained inherent antioxidant activity of the drug	[69]

## Data Availability

Not applicable.
